# Ciprofol injection in patients receiving non-invasive positive pressure ventilation: a retrospective study

**DOI:** 10.3389/fphar.2026.1745150

**Published:** 2026-02-24

**Authors:** DaLong Zhang, Lan Xiao, Xue Lv, ChenXi Ma, XiaoHan Zhang, XingGuo Niu

**Affiliations:** The Fifth Clinical Medical College of Henan University of Chinese Medicine (Zhengzhou People’s Hospital), Zhengzhou, Henan, China

**Keywords:** ciprofol, non-invasive positive pressure, retrospective study, safety, sedation

## Abstract

**Object:**

This study aims to compare the efficacy and safety of ciprofol versus propofol in sedation for patients undergoing non-invasive positive pressure ventilation (NPPV), with a focus on evaluating sedative efficiency and systematically comparing the incidence of respiratory depression, circulatory depression, and adverse reactions.

**Methods:**

A retrospective analysis was conducted on the clinical data of 120 patients receiving NPPV at our hospital between June 2024 and October 2025. According to the sedative agent used, patients were divided into a Ciprofol group (Group C, n = 56) and a Propofol group (Group P, n = 64). The primary outcome was the sedation induction time, defined as the duration from drug administration to the first achievement of the target Richmond Agitation-Sedation Scale (RASS) score of −2 to 0. Secondary outcomes included the recovery time, extubation time, dynamic changes in respiratory rate, peripheral oxygen saturation (SpO_2_), and mean arterial pressure (MAP) during treatment, as well as the incidence of adverse reactions such as hypoxemia (SpO_2_ < 90%), hypotension, and injection pain.

**Results:**

Regarding the primary outcome, the sedation induction time was comparable between the two groups, with no statistically significant difference (p > 0.05). Analysis of secondary outcomes revealed that the Ciprofol group demonstrated several advantages: ① Enhanced Safety Profile: The incidences of hypoxemia and hypotension were significantly lower in the Ciprofol group (p < 0.05). ② Faster Recovery: Both emergence time and full recovery time were shorter in the Ciprofol group compared to the Propofol group (p < 0.05). ③ Improved Tolerability: The incidence of injection pain was markedly lower in the Ciprofol group (p < 0.001), and fluctuations in respiratory and circulatory parameters during treatment were also reduced.

**Conclusion:**

For patients undergoing NPPV, ciprofol provides sedation efficacy comparable to propofol while demonstrating a superior safety profile, faster recovery, and enhanced injection tolerability. This combination of advantages makes ciprofol a preferable alternative for sedation in this clinical setting.

## 1 Introduction

Non-invasive Positive-Pressure Ventilation (NPPV) is a commonly used treatment for acute respiratory failure, widely applied in various clinical scenarios such as acute exacerbations of chronic obstructive pulmonary disease, cardiogenic pulmonary edema, and post-extubation respiratory support (Berbe et al., 2020; Lightowler et al., 2003; Chawla et al., 2020). However, during NPPV therapy, patients often experience anxiety, agitation, and patient-ventilator asynchrony—commonly referred to as “Patient-Ventilator Asynchrony”—due to factors including the underlying disease, unfamiliar surroundings, and intolerance to the ventilator (Carrillo et al., 2012). This phenomenon involves various physiological mechanisms such as ineffective triggering and double triggering, and is significantly associated with adverse outcomes including increased patient mortality and prolonged duration of ventilation (L et al., 2015). It not only increases patients’ oxygen consumption and exacerbates organ hypoxia but may also reduce ventilation effectiveness, potentially leading to treatment interruption or escalation to endotracheal intubation. Therefore, analgesia and sedation have become critical strategies for improving NIV tolerance and synchronization (Devlin et al., 2018). Currently, drugs commonly used for sedation during NPPV in clinical practice include propofol and dexmedetomidine, but risks such as respiratory depression and hemodynamic fluctuations still exist (Buckley et al., 2020). Ciprofol, a novel GABA-A receptor agonist, exhibits rapid onset, fast metabolism, and controllable depth of sedation. In recent years, it has demonstrated favorable safety and efficacy in general anesthesia and procedural sedation (Liu et al., 2024; Teng et al., 2021; Liu et al., 2023). However, direct comparative evidence regarding its value in sedation for NPPV patients remains lacking (Yang et al., 2024). Therefore, this retrospective analysis aims to systematically compare the sedative efficacy and safety of ciprofol versus propofol in NPPV patients, providing a reference for rational selection of sedative agents in this population.

## 2 Methods

### 2.1 Study design and population

**FIGURE 1:**
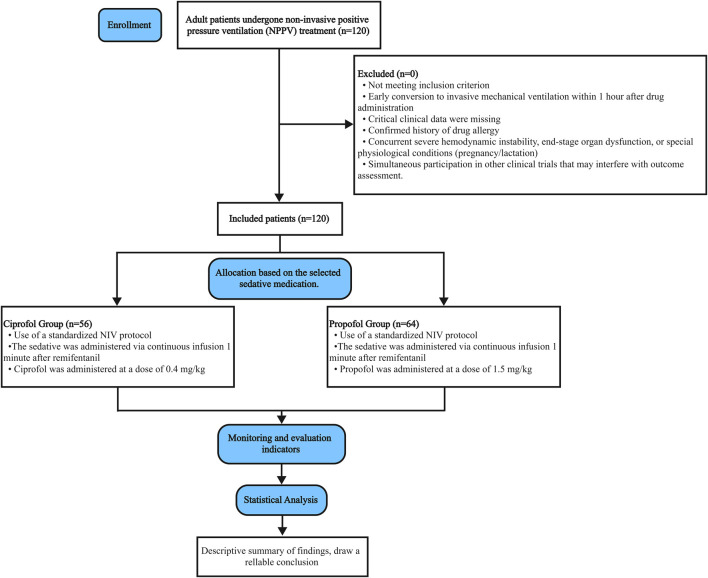
Flowchart of patient enrollment and group allocation.

This study employed a single-center retrospective cohort design to systematically screen and analyze the complete clinical records of 120 patients who received Non-invasive Positive-Pressure Ventilation (NPPV) treatment at our hospital between January 2024 and October 2025. Patients were divided into two groups based on sedative selection: Group C (ciprofol group, n = 56) and Group P (propofol group, n = 64). All patients received NPPV support for acute respiratory failure and required sedation to alleviate patient-ventilator asynchrony. Through strict matching of baseline characteristics, the two groups were comparable with no significant differences in age, gender, BMI, or distribution of underlying diseases (P > 0.05). The detailed process of study screening is depicted in Figure 1.

### 2.2 Inclusion and exclusion criteria

Researchers developed multidimensional inclusion and exclusion criteria to ensure data quality. Inclusion criteria include: ① Adult patients (≥18 years old); ② Confirmed acute respiratory failure receiving non-invasive ventilation support; ③ Complete documentation of sedation treatment using ciprofol or propofol; ④ RASS score ≥ +1 prior to medication administration, with agitation requiring sedation; ⑤ Complete medical record documentation, including baseline vital signs, vasoactive drug usage, and hepatic/renal function laboratory data. Exclusion criteria include: ① Early conversion to invasive ventilation (within 1 h of drug administration); ② Missing key clinical data (e.g., RASS score, vital signs); ③ Confirmed history of drug allergy; ④ Concurrent severe hemodynamic instability, end-stage organ dysfunction, or special physiological conditions (pregnancy/lactation); ⑤ Simultaneous participation in other clinical trials that may interfere with outcome assessment.

### 2.3 Intervention protocol

All patients received standardized non-invasive ventilation support, with ventilator parameters dynamically adjusted based on arterial blood gas analysis results. A unified analgesia and sedation protocol was implemented: first, all patients received continuous infusion of remifentanil as background analgesia. Remifentanil was prepared by diluting 2 mg in 50 mL of normal saline (final concentration 40 μg/mL), with an initial infusion rate set at 3–6 mL/h (approximately equivalent to 0.0017–0.0034 mg/kg/h). One minute after the start of remifentanil infusion, a loading dose of the sedative drug was administered intravenously—patients in the C group received ciprofol 0.4 mg/kg, while those in the P group received propofol 1.5 mg/kg. Maintenance infusion of the sedative was initiated immediately thereafter. Both ciprofol and propofol were administered as ready-to-use intravenous solutions. In this study, ciprofol was used at a concentration of 2.5 mg/mL, and propofol at 10 mg/mL. Based on the drug characteristics and individual patient response, the infusion rates were individually titrated: the initial maintenance infusion rate for ciprofol in the C group was typically 2–8 mL/h (approximately equivalent to 0.05–0.20 mg/kg/h), and for propofol in the P group, 2–12 mL/h (approximately equivalent to 0.20–1.20 mg/kg/h). The infusion rates of all drugs were dynamically titrated according to patient response, with the ultimate goal of maintaining the patient’s Richmond Agitation-Sedation Scale (RASS) score between −2 and 0. Sedation depth, respiratory and circulatory parameters, and adverse reactions were continuously monitored throughout the treatment period.

### 2.4 Outcome measures

The primary outcome focused on sedation efficacy and was assessed using commonly adopted ICU standards: sedation induction time (defined as the time from the initiation of the study drug infusion to the first time the patient achieved the target sedation depth). Sedation depth was evaluated using the Richmond Agitation-Sedation Scale (RASS), a validated and reliable tool for assessing consciousness and sedation levels in ICU patients. The RASS score ranges from −5 (unarousable) to +4 (combative), with 0 representing an alert and calm state (Cn et al., 2002). In this study, the target sedation depth was defined as a RASS score between −2 and 0.

Secondary outcomes encompassed a multidimensional evaluation, including: ① Safety indicators: the incidence of hypoxemia (defined as SpO_2_ <90% for >1 min) and hypotensive events (defined as a mean arterial pressure [MAP] <65 mmHg or a decrease of >30% from baseline); ② Recovery quality indicators: time to awakening (from discontinuation of sedative infusion until RASS score ≥ −1) and time to full recovery (until RASS score returned to 0); ③ Respiratory function indicators: the temporal changes in respiratory rate (RR), oxygen saturation (SpO_2_), oxygenation index (PaO_2_/FiO_2_), and partial pressure of carbon dioxide (PaCO_2_); ④ Hemodynamic indicators: the temporal changes in mean arterial pressure (MAP) and heart rate (HR); ⑤ Other adverse reactions: the incidence of bradycardia, injection pain, nausea/vomiting, and delirium (defined by an Intensive Care Delirium Screening Checklist [ICDSC] score ≥4). Data for all time-sequenced indicators were collected at four fixed time points: before sedation initiation (baseline, T1), and at 1 h (T2), 6 h (T3), and 24 h (T4) after initiation.

### 2.5 Statistical analysis

As a retrospective analysis, the sample size of this study was constrained by the availability of case data within a specific timeframe; therefore, a formal prospective *a priori* sample size calculation was not performed. To evaluate the statistical power of the current sample size for detecting a difference in the primary outcome (sedation induction time), a simulation-based estimation was conducted.

The parameters for this simulation were set as follows: a between-group difference of 5 s, which was defined as the prespecified “minimum clinically important difference” for this study—a standardized approach used to determine whether an observed difference in clinical outcomes holds practical significance (Ja et al., 2021); and a standard deviation of 10 s, serving as a conservative estimate of variability during the induction phase. The 5-s threshold was informed by evidence from a recent systematic review indicating no statistically significant difference in sedation induction time between ciprofol and propofol (Armengo et al., 2026). The 10-s standard deviation was contextualized against the considerably larger inter-individual variability (standard deviation of approximately 390 s) reported in a study of a similar ICU population (Liu et al., 2023); the use of a substantially smaller value in our simulation aimed to establish a stringent testing scenario, demonstrating that the sample size would remain adequate even under assumptions that underestimate variability.

Based on these conservative parameters, the simulated required sample size was in the same order of magnitude and closely aligned with the 120 patients actually included in this study. This indicates that, even under the strictest assumptions, the present sample size provides reasonable and acceptable statistical power for detecting the prespecified difference in the primary outcome.

This study employed SPSS 27.0 for statistical analysis. Quantitative data were verified as normally distributed via the Shapiro-Wilk test and described as mean ± standard deviation (x ± s). Intergroup comparisons were performed using the independent samples t-test. Qualitative data were expressed as frequency (percentage), with intergroup comparisons conducted using the chi-square test. All tests were two-tailed, with *p* < 0.05 indicating statistically significant differences.

## 3 Results

### 3.1 Participant characteristics and flow diagram

A total of 120 patients undergoing treatment with NPPV were included in the study. The cohort comprised 72 males and 48 females, with a mean age of 48.0 ± 14.8 years and a mean body mass index (BMI) of 18.5 ± 3.1 kg/m^2^. There were no significant differences in general characteristics such as age, gender, and BMI between the two groups (*p* > 0.05), indicating comparability (Table 1).

**TABLE 1:** Comparison of patient baseline characteristics (n = 120, $\bar{x}$ ± s, [n (%)]).

Characteristics	Ciprofol (n = 56)	Propofol (n = 64)	χ ^2^/*t* value	*p* value
Gender [n (%)]				
Male	31 (43.06)	41 (56.94)	0.943^1)^	0.331
Female	25 (52.08)	23 (47.92)		
Age	48.1 ± 14.5	47.9 ± 15.5	0.306^2)^	−0.208
BMI/(kg/m^2^)	18.4 ± 3.5	18.6 ± 2.9	−0.208^2)^	0.835
Medical history [n (%)]				
Acute exacerbation of chronic obstructive pulmonary disease	16 (55.17)	13 (44.83)	1.112^1)^	0.292
Cardiac pulmonary edema	21 (48.84)	22 (51.16)	0.127^1)^	0.722
Respiratory support after extubation	5 (50.0)	5 (50.0)	0.049^1)^	0.825
Mild to moderate ARDS	18 (47.37)	20 (52.63)	0.011^1)^	0.916

1. Represents the chi-square value (χ^2^); 2. Represents the *t*-value.

### 3.2 Primary outcomes

The primary outcomes for both groups are presented in Table 2. Regarding sedation induction, there was no statistically significant difference in the induction time to achieve the target sedation depth (RASS -2 to 0) between the C group and the P group (t = −1.256, p = 0.212). This result indicates that when used for sedation in NPPV patients, ciprofol has a comparable speed of onset to propofol, and the two drugs show similar efficacy in terms of initial sedative effect.

**TABLE 2:** Comparison of sedation induction time between the two groups ( $\bar{x}$ ± s).

Parameter	Ciprofol (n = 56)	Propofol (n = 64)	*t* value	*p* value
Sedation induction time(s)	70.2 ± 11.4	73.5 ± 16.5	−1.256	0.212

Sedation induction time was defined as the duration from the initiation of the study drug infusion to the first time the patient achieved the target sedation depth. Sedation depth was assessed using the Richmond Agitation-Sedation Scale (RASS), with a target range of −2, to 0.

### 3.3 Secondary outcomes

#### 3.3.1 Safety outcomes

**FIGURE 2:**
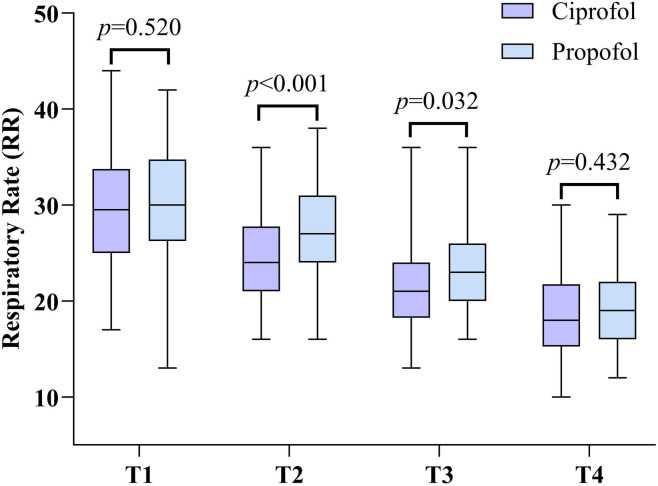
Respiratory Rate (RR) at different time points in the ciprofol and propofol groups.

The safety comparison between the two patient groups is shown in Tables 3, 4. In terms of respiratory depression, the incidence of hypoxemia was significantly lower in group C than in group P (χ^2^ = 4.293, *p* = 0.038). Regarding circulatory depression, the frequency of hypotension was also significantly lower in group C compared to group P (χ^2^ = 5.198, *p* = 0.023). Dynamic monitoring of RR (Table 3) further corroborated these findings. No significant difference in RR was observed between groups at baseline (T1) (*p* > 0.05). However, at T2 and T3, RR was significantly higher in group P than in group C. By T4, the difference in RR had no statistical significance (*p* > 0.05) (Figure 2).

**TABLE 3:** Incidence of hypoxemia (SpO_2_ <90%) and hypotension [n (%)].

Parameter	Ciprofol (n = 56)	Propofol (n = 64)	χ ^2^value	*p* value
SpO2<90%	6 (10.71)	15 (23.44)	4.293	0.038
Hypotension	7 (12.50)	19 (29.69)	5.198	0.023

**TABLE 4:** Comparison of respiratory rates between the two groups **(** $\bar{x}$ ± s**)**.

Parameter	Time point	Ciprofol (n = 56)	Propofol (n = 64)	*t* value	*p* value
Respiratory rate (RR)	T1	29.5 ± 6.5	30.2 ± 5.7	−0.646	0.520
	T2	24.2 ± 4.6	27.4 ± 5.0	−3.572	<0.001
	T3	21.8 ± 4.8	23.6 ± 4.5	−2.174	0.032
	T4	18.8 ± 4.6	19.4 ± 3.8	−0.789	0.432

#### 3.3.2 Recovery characteristics

**FIGURE 3:**
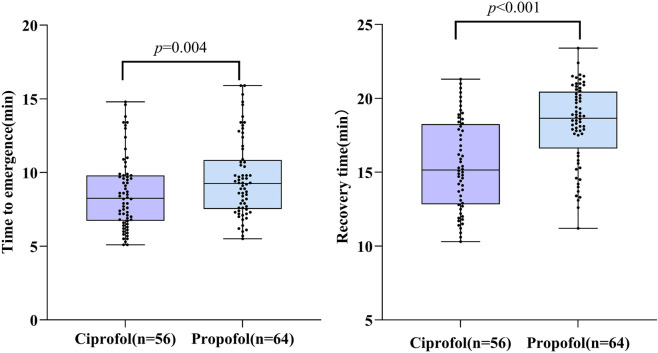
Comparison of time to emergence and recovery time between ciprofol group and propofol group.

During the recovery phase, the C group demonstrated significant advantages. Compared with the P group (propofol group), patients in the C group had a significantly shorter time to awakening (to RASS ≥ −1) (C group: 8.3 ± 2.3 min vs. P group: 9.6 ± 2.7 min; t = −2.967, p = 0.004). Furthermore, the time to full recovery (to RASS = 0) was also significantly shorter in the C group than in the P group (C group: 15.5 ± 3.1 min vs. P group: 18.3 ± 2.7 min; t = −5.264, p < 0.001). These results indicate that after achieving an equivalent sedative effect, ciprofol promotes faster and more stable emergence and recovery in patients. (Table 5) (Figure 3).

**TABLE 5:** Comparison of recovery quality between the two groups ( $\bar{x}$ ± s).

Recovery parameter	Ciprofol (n = 56)	Propofol (n = 64)	t value	*p* value
Emergence time (min)	8.3 ± 2.3	9.6 ± 2.7	−2.967	0.004
Recovery time (min)	15.5 ± 3.1	18.3 ± 2.7	−5.264	<0.001

Recovery time was defined as the time from discontinuation of sedative infusion until the patient’s RASS, score reached ≥ −1, and full recovery time was defined as the time from discontinuation of sedative infusion until the patient’s RASS, score returned to 0 (awake and calm).

#### 3.3.3 Respiratory effects

No statistically significant differences were detected between the two groups in SpO_2_, PaO_2_/FiO_2_, and PaCO_2_ levels at T1 (p > 0.05). SpO_2_ and PaO_2_/FiO_2_ levels increased gradually during continuous intravenous infusion of ciprofol and propofol in group C and group P. Meanwhile, PaCO2 levels peaked at T2 and then gradually decreased in both groups. At T2 and T3, patients in group C had significantly higher SpO_2_ and PaO_2_/FiO_2_ levels than those in group P (p < 0.05), along with lower PaCO2 levels (p < 0.05). Notably, by T4, there was no statistically significant difference in these respiratory measures between the groups (p > 0.05) (Table 6).

**TABLE 6:** Comparison of respiratory parameters between the two groups ( $\bar{x}$ ± s).

Parameter	Time point	Ciprofol (n = 56)	Propofol (n = 64)	*t* value	*p* value
SpO_2_(%)	T1	80.7 ± 5.2	80.1 ± 4.8	0.691	0.491
	T2	88.5 ± 3.3	86.3 ± 3.6	3.464	<0.001
	T3	93.0 ± 2.5	91.8 ± 3.1	0.365	0.023
	T4	95.7 ± 1.8	95.2 ± 2.6	1.257	0.211
PaO2/FiO2	T1	210.9 ± 21.2	207.9 ± 34.1	0.571	0.569
	T2	257.7 ± 13.1	242.0 ± 14.6	6.169	<0.001
	T3	289.2 ± 19.7	280.5 ± 19.9	2.413	0.017
	T4	310.8 ± 18.0	305.8 ± 19.2	1.467	0.145
PaCO_2_ (mmHg)	T1	49.5 ± 3.4	50.1 ± 4.4	−0.789	0.432
	T2	51.7 ± 4.7	54.8 ± 3.7	−4.163	<0.001
	T3	48.9 ± 3.1	50.0 ± 1.9	−2.264	0.025
	T4	45.9 ± 3.8	46.9 ± 3.4	−1.502	0.136

#### 3.3.4 Hemodynamic effects

There was no significant difference in MAP and HR between the two groups at T1 (*p* > 0.05). However, at T2, both MAP and HR were significantly higher in group C compared to group P (*p* < 0.001). These differences persisted at T3, with MAP and HR remaining elevated in group C (*p* = 0.025, *p* = 0.012). By T4, no significant difference was observed, similar to T1 (*p* > 0.05) (Table 7).

**TABLE 7:** Comparison of hemodynamic parameters between the two groups ( $\bar{x}$ ± s).

Parameter	Time point	Ciprofol (n = 56)	Propofol (n = 64)	*t* value	*p* value
MAP (mmHg)	T1	90.4 ± 7.3	90.9 ± 7.8	−0.395	0.694
	T2	82.5 ± 9.8	70.9 ± 10.4	6.258	<0.001
	T3	86.4 ± 9.8	82.2 ± 9.5	2.263	0.025
	T4	86.6 ± 9.6	85.2 ± 8.6	0.840	0.402
HR	T1	82.2 ± 9.7	82.9 ± 9.2	−0.441	0.660
	T2	78.2 ± 9.1	70.9 ± 9.2	4.322	<0.001
	T3	82.0 ± 8.3	78.1 ± 8.1	2.559	0.012
	T4	82.2 ± 8.8	79.5 ± 9.3	1.588	0.115

#### 3.3.5 Adverse events

In this study, 26.79% (15 of 56) of patients in group C experienced adverse events. No serious adverse events occurred, and no participants discontinued the study due to adverse events. All reported adverse events were resolved after treatment. In group C, adverse events included bradycardia (3 patients, 5.36%), injection pain (1 patient, 1.79%), nausea/vomiting (5 patients, 8.93%), and delirium (6 patients, 10.71%).

At the same time, 70.31% (45 of 64) of patients in group C suffered adverse events. No significant adverse events occurred, and no participants discontinued the study due to adverse events. All reported adverse events were resolved after treatment. Adverse events in group P included bradycardia (7 patients, 10.94%), injection pain (18 patients, 28.13%), nausea/vomiting (11 patients, 17.19%), and delirium (9 patients, 14.06%).

**FIGURE 4:**
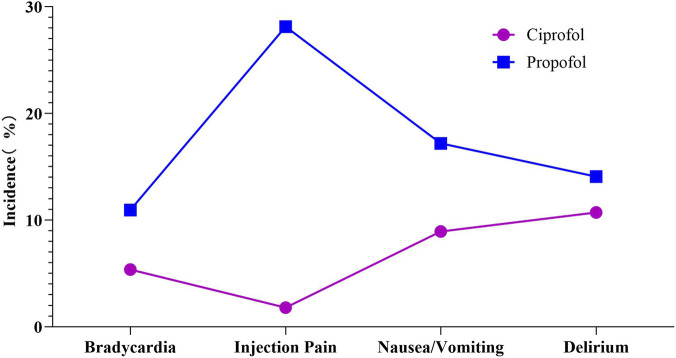
Comparison of adverse events between the ciprofol group and propofol group.

The incidence of injection pain was significantly higher in group P compared to group C, demonstrating a statistically significant difference (χ^2^ = 15.548, *p* < 0.001). However, no significant differences were observed in other adverse events between group C and group P (*p* > 0.05) (Table 8; Figure 4).

**TABLE 8:** Comparison of adverse drug reactions between the two group**s** [n (%)].

Adverse reaction	Ciprofol (n = 56)	Propofol (n = 64)	χ ^2^ value	*p* value
Bradycardia	3 (5.36)	7 (10.94)	1.218	0.270
Injection pain	1 (1.79)	18 (28.13)	15.548	<0.001
Nausea/Vomiting	5 (8.93)	11 (17.19)	1.763	0.184
Delirium	6 (10.71)	9 (14.06)	0.306	0.580

Delirium (ICDSC, score ≥4).

## 4 Discussion

Non-invasive Positive-Pressure Ventilation (NPPV) is widely used in clinical practice for patients with respiratory failure of various reasons. However, many patients experience non-physiological states such as pain, tension, anxiety, and agitation during treatment, which can lead to the phenomenon of “human-machine conflict” (Ryu et al., 2022). Such conflict can be severe enough to endanger the patient’s life. Therefore, sedation plays a critical role in the management of these patients. It alleviates anxiety, reduces excessive oxygen consumption, and thus improves patient-ventilator synchrony to ensure treatment efficacy (Cammarota et al., 2022). However, it is necessary to pay attention to the impact of sedative drugs on hemodynamics and the inhibition of respiratory.

### 4.1 Main finding

This retrospective cohort study systematically compared the performance of ciprofol versus propofol for sedation in patients receiving non-invasive positive pressure ventilation. The core findings demonstrate that ciprofol provides sedation equally effective to propofol, as evidenced by no significant difference in induction time. Building on this equivalent efficacy, ciprofol exhibited significant advantages in safety and recovery quality: it was associated with a significantly lower incidence of both hypoxemia and hypotension, shorter times to awakening and full recovery, and a markedly reduced incidence of injection pain. Collectively, these results suggest that ciprofol is an effective and safer alternative for sedation during NPPV therapy.

### 4.2 Relation with previous evidence

The findings of this study corroborate previous research and provide important additional evidence regarding the safety advantages of ciprofol within a real-world clinical setting. The relevance of our results to existing evidence is analyzed below from several perspectives.

#### 4.2.1 Sedative efficiency

This study confirms that there is no significant difference in sedation induction time between propofol and propofol, consistent with their shared pharmacological mechanism as GABA-A receptor agonists (Lu et al., 2023; Akhtar et al., 2024; Nair and Seelam, 2022). This finding establishes a primary foundation for ciprofol as an equivalent alternative to propofol in NPPV scenarios, indicating their comparable efficacy in achieving initial sedation targets.

#### 4.2.2 Quality of recovery

Beyond demonstrating equivalent sedative efficacy, this study further reveals the superior recovery profile associated with ciprofol. The results indicate that the ciprofol group exhibited significantly shorter emergence time and recovery time, which is closely related to its more rapid metabolic clearance and shorter plasma elimination half-life (Lu et al., 2023). This pharmacokinetic profile holds significant implications for the ICU setting, where rapid neurological assessment and minimizing cumulative sedative effects are crucial.

#### 4.2.3 Respiratory and circulatory safety

Regarding the critical dimension of safety, the findings of this study reinforce the superior profile of ciprofol. Previous research has indicated a significant risk of respiratory depression associated with propofol (Hu et al., 2022). This study substantiates inferences from prior phase I-III clinical trials (Teng et al., 2021; Liu et al., 2021; Liu et al., 2022), demonstrating that in real-world clinical settings, ciprofol causes less pronounced respiratory depression: the incidence of hypoxemia was significantly lower in the ciprofol group compared to the propofol group. Furthermore, dynamic monitoring data revealed that during the critical post-sedation period (time points T2 and T3), patients in the ciprofol group had significantly better oxygenation indices (SpO_2_, PaO_2_/FiO_2_) and significantly lower PaCO_2_ levels. The advantage of ciprofol in maintaining efficient gas exchange was particularly prominent during the critical 1–6 h period following administration. Notably, most patients who experienced hypoxemia had moderate ARDS. This may be related to the ARDS-induced damage to the alveolar-capillary membrane, increased permeability, pulmonary edema, and atelectasis, which predispose patients to severe baseline hypoxemia and hypercapnia (Hussain et al., 2021; Chen et al., 2020). This suggests that underlying pulmonary function may amplify the differential respiratory depressant effects of sedative agents.

Similarly, regarding circulatory stability, the ciprofol group had a significantly lower incidence of hypotension and a smaller magnitude of blood pressure decrease. This phenomenon may be attributable to ciprofol’s lesser impact on vascular tone and myocardial contractility (Wei et al., 2022). It is particularly noteworthy that although heart rate in the ciprofol group decreased to some extent during anesthesia, the degree of fluctuation was significantly smaller than in the propofol group, indicating superior hemodynamic stability. This finding provides clinical evidence for the circulatory safety of ciprofol, consistent with previous basic research results (Zhong et al., 2023).

Collectively, these findings demonstrate that ciprofol achieves the target sedative effect while causing less perturbation to the respiratory and circulatory systems, indicating a more favorable clinical safety profile.

#### 4.2.4 Tolerability and other adverse reactions

Injection pain is one of the most prominent adverse reactions associated with propofol. The concentration of propofol is a key factor contributing to injection pain, which can cause discomfort, increase patient distress and anxiety, and trigger physical movements that interfere with the smooth implementation of noninvasive mechanical ventilation. This study observed a significantly lower incidence of injection pain in the ciprofol group compared to the propofol group (1.79% vs. 28.13%), a finding of considerable clinical significance. From a pharmacological perspective, this difference may stem from ciprofol’s greater hydrophobicity and lower plasma concentration, resulting in reduced aqueous phase concentration and consequently diminished vascular irritation (Chen et al., 2022). Additionally, regarding other adverse reactions, there were no significant differences in the incidence of bradycardia, nausea/vomiting, or similar events between the two groups. These reactions were transient in nature and resolved rapidly following treatment, consistent with the safety profile of similar sedative medications (Buckley et al., 2020).

### 4.3 Strengths and limitations

Our study demonstrates the efficacy and safety of ciprofol for sedation during NPPV. However, several limitations should be considered. First, the retrospective design may not fully preclude confounding factors despite strict inclusion and exclusion criteria. Second, the sample size, though adequate for the primary outcome as contextualized by our simulation (which employed conservative prespecified parameters: a 5-s minimal clinically important difference and a 10-s standard deviation), remains relatively limited. This may constrain the statistical power to detect differences in rare adverse events. Third, the observation period was confined to the sedation and immediate recovery phases, precluding evaluation of long-term outcomes. Finally, although sedation induction time as the primary endpoint directly reflects the drug’s onset of action, future studies could employ more comprehensive metrics—such as time to stable sedation or NPPV tolerance scores—to holistically assess sedation quality.

These limitations highlight the necessity for validating the current conclusions through prospective, multicenter, randomized controlled trials with larger sample sizes. Beyond confirming clinical efficacy, future research should also incorporate pharmacoeconomic evaluations, which are critical for optimizing resource allocation in the high-cost intensive care unit setting (Ss et al., 2012).

## 5 Conclusion

In summary, this study confirms that in patients receiving NPPV, ciprofol provides sedation comparable in efficacy to propofol while offering a comprehensive superior profile characterized by mitigated respiratory and circulatory inhibition, expedited recovery, and a lower incidence of injection pain. These findings provide an important basis for clinicians to select a safer sedation strategy for this vulnerable population. Future research should validate these findings through prospective designs and explore its optimal application in specific patient subgroups.

## Data Availability

The original contributions presented in the study are included in the article/supplementary material, further inquiries can be directed to the corresponding author.
